# Learning curves of resection and reconstruction procedures in robotic-assisted pancreatoduodenectomy by a single surgeon: a retrospective cohort study of 160 consecutive cases

**DOI:** 10.1093/gastro/goad042

**Published:** 2023-07-26

**Authors:** Xi-Tai Huang, Xi-Yu Wang, Jin-Zhao Xie, Jian-Peng Cai, Wei Chen, Liu-Hua Chen, Xiao-Yu Yin

**Affiliations:** Department of Pancreato-Biliary Surgery, The First Affiliated Hospital, Sun Yat-sen University, Guangzhou, Guangdong, P. R. China; Department of Pancreato-Biliary Surgery, The First Affiliated Hospital, Sun Yat-sen University, Guangzhou, Guangdong, P. R. China; Department of Pancreato-Biliary Surgery, The First Affiliated Hospital, Sun Yat-sen University, Guangzhou, Guangdong, P. R. China; Department of Pancreato-Biliary Surgery, The First Affiliated Hospital, Sun Yat-sen University, Guangzhou, Guangdong, P. R. China; Department of Pancreato-Biliary Surgery, The First Affiliated Hospital, Sun Yat-sen University, Guangzhou, Guangdong, P. R. China; Department of Pancreato-Biliary Surgery, The First Affiliated Hospital, Sun Yat-sen University, Guangzhou, Guangdong, P. R. China; Department of Pancreato-Biliary Surgery, The First Affiliated Hospital, Sun Yat-sen University, Guangzhou, Guangdong, P. R. China

**Keywords:** robotic-assisted surgery, pancreatoduodenectomy, learning curve, outcomes

## Abstract

**Background:**

Robotic-assisted pancreatoduodenectomy (RPD) has been routinely performed in a few of centers worldwide. This study aimed to evaluate the perioperative outcomes and the learning curves of resection and reconstruction procedures in RPD by one single surgeon.

**Methods:**

Consecutive patients undergoing RPD by a single surgeon at the First Affiliated Hospital of Sun Yat-sen University (Guangzhou, China) between July 2016 and October 2022 were included. The perioperative outcomes and learning curves were retrospectively analysed by using cumulative sum (CUSUM) analyses.

**Results:**

One-hundred and sixty patients were included. According to the CUSUM curve, the times of resection and reconstruction procedures were shortened significantly after 30 cases (median, 284 vs 195 min; *P *<* *0.001) and 45 cases (median, 138 vs 120 min; *P *<* *0.001), respectively. The estimated intraoperative blood loss (median, 100 vs 50 mL; *P *<* *0.001) and the incidence of clinically relevant post-operative pancreatic fistula (29.2% vs 12.5%; *P *=* *0.035) decreased significantly after 20 and 120 cases, respectively. There were no significant differences in the total number of lymph nodes examined, post-operative major complications, or post-operative length-of-stay between the two groups.

**Conclusions:**

Optimization of the resection procedure and the acquisition of visual feedback facilitated the performance of RPD. RPD was a safe and feasible procedure in the selected patients.

## Introduction

Pancreatoduodenectomy (PD) is one of the most troublesome procedures due to its technically demanding nature and high post-operative morbidities of ∼40%–50% [1–3]. Clinically relevant post-operative pancreatic fistula (CR-POPF) is one of the most common complications after PD, which can lead to lethal sequelae, including severe intra-abdominal infection, intra-abdominal hemorrhage, and even death [4].

With the development of the robotic-assisted surgical system, robotic-assisted pancreatoduodenectomy (RPD) has emerged as an alternative to laparoscopic pancreatoduodenectomy (LPD) and open pancreatoduodenectomy (OPD) [5]. Armed with 3D vision and improved dexterity, RPD is theoretically more flexible and stable than conventional OPD or LPD [6, 7]. Previous studies showed that RPD could reduce the intraoperative blood loss and post-operative hospital stay compared with OPD [8, 9]. However, due to the lack of force feedback in a robotic-assisted surgical system, the surgeon needs to overcome this limitation by finishing a certain number of cases. Unskilled operators may increase the operation time and the risk of post-operative complications.

To date, only a few of pancreatic centers have reported more than 150 cases of RPD worldwide [9–13]. Among them, only four studies reported the learning curve of RPD with more than 150 cases in a single center [9–12]. These studies had some limitations. The RPD procedures in these studies were mostly performed by several surgeons, which will have inevitable bias on analysis of the learning curve. On the other hand, RPD was a multi-step operation, consisting of resection and reconstruction procedures, which might have different learning curves. Previous studies about the learning curve of RPD only focused on the total operative time and so far no separate analyses of learning curves of resection and reconstruction procedures in RPD have been reported. The present study aimed to separately evaluate the learning curves of resection and reconstruction procedures in RPD by a single surgeon, which would be valuable to optimize the surgical procedures to reduce the operation time and post-operative complications.

## Materials and methods

### Patient selection

Consecutive patients who underwent RPD by a single surgeon at the First Affiliated Hospital of Sun Yat-sen University (Guangzhou, China) between July 2016 and October 2022 were included in this study.

**Figure 1.**
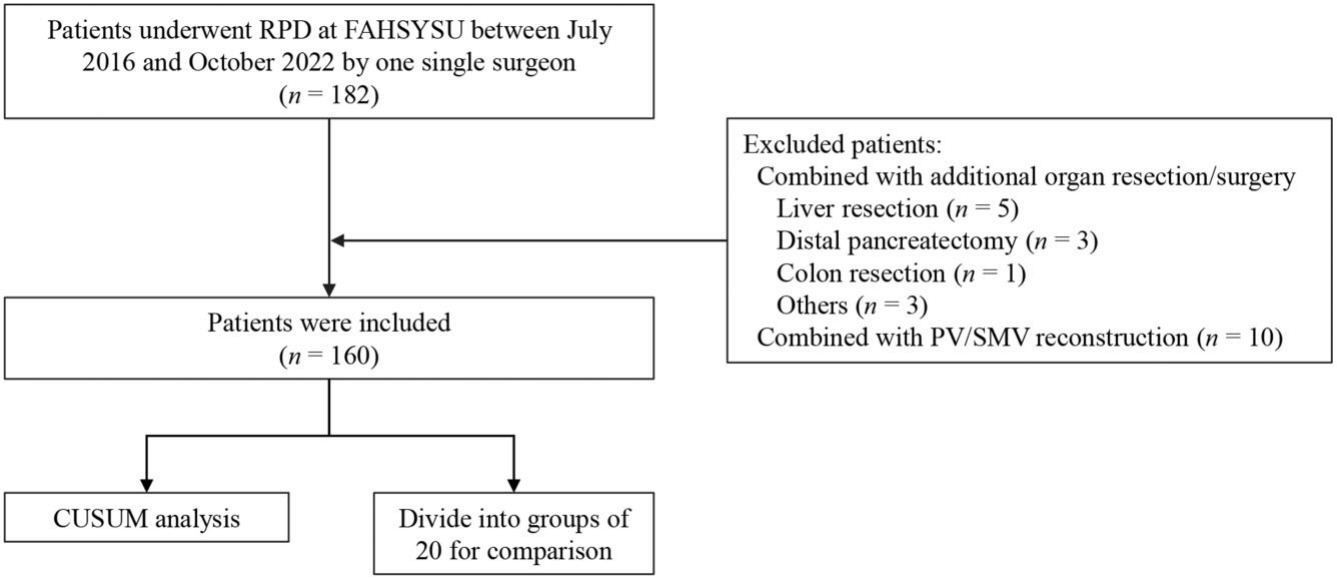
Flow chart of patient selection in this study. RPD, robotic-assisted pancreatoduodenectomy; FAHSYSU, First Affiliated Hospital of Sun Yat-sen University; CUSUM, cumulative sum; PV, portal vein; SMV, superior mesenteric vein.

The exclusion criteria included: (i) combined with additional organ resection; and (ii) combined with vascular resection and reconstruction, including portal vein (PV) or superior mesenteric vein (SMV). Finally, 160 patients were enrolled in this study and the flow chart of the patient selection is presented in Figure 1. This study was approved by the Ethics Committee of the First Affiliated Hospital of Sun Yat-sen University (Approval number: [2022]025).

### Surgical technique

**Figure 2.**
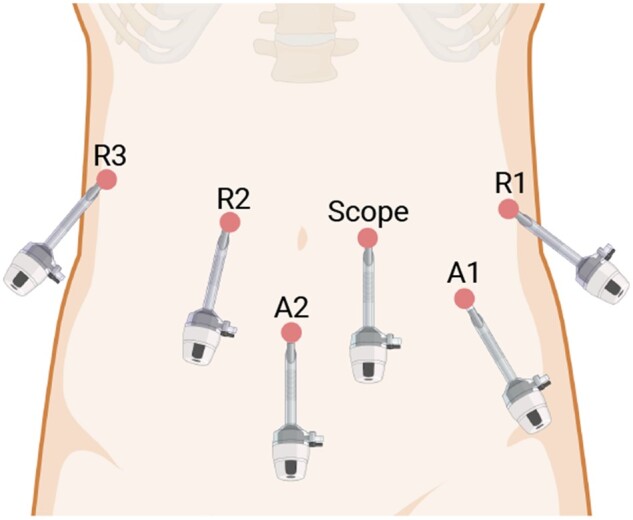
Trocar placement for robotic-assisted pancreatoduodenectomy. R1, 8-mm trocar for the first robotic arm; R2, 8-mm trocar for the second robotic arm; R3, 8-mm trocar for the third robotic arm; A1-A2, 12-mm trocars for assistant instruments.

All RPD procedures were performed by one single surgeon (Prof. Xiao-Yu Yin) by using a da Vinci Si Surgical System (Intuitive Surgical, Inc., Sunnyvale, CA, USA) with four articulating robotic arms: a central arm for a 30° rigid endoscope, a first arm for permanent cautery hook or Harmonic ACE curved shears or needle driver, a second arm for fenestrated bipolar forceps, and a third arm for Cadiere forceps. Two assistant’s ports were placed. The trocar placement is present in Figure 2.

### Operative procedures of RPD (“nine-step” procedures)

**Figure 3.**
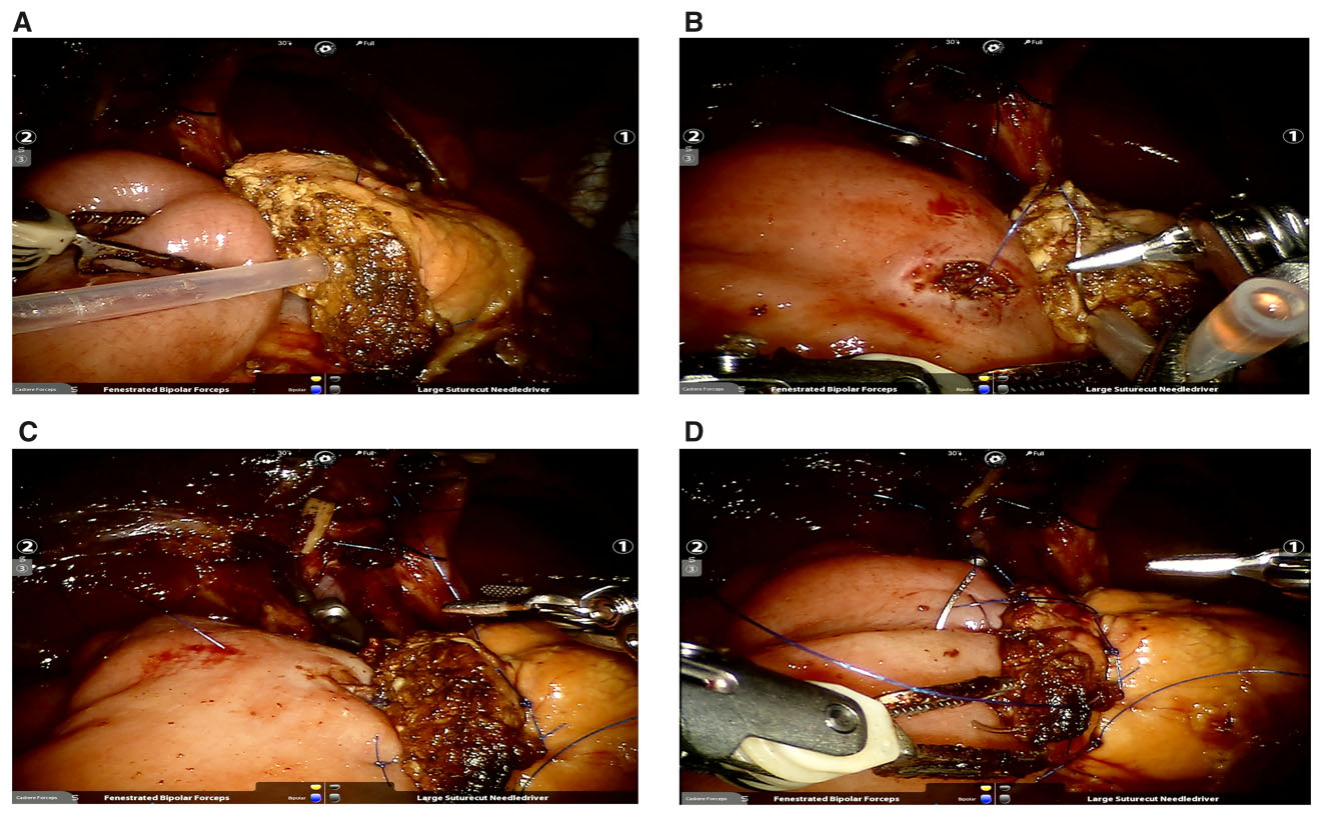
The procedure of end-to-side mucosa-to-mucosa pancreatojejunostomy. (A) The posterior external wall is anastomosed by three overlapping interrupted transpancreatic U-sutures. (B) The posterior internal wall is anastomosed by interrupted suture of the pancreatic ductal mucosa and jejunal mucosa. (C) The anterior internal wall is anastomosed by interrupted suture of the pancreatic ductal mucosa and jejunal mucosa. (D) Anterior external wall is anastomosed by continuous suture.

**Figure 4.**
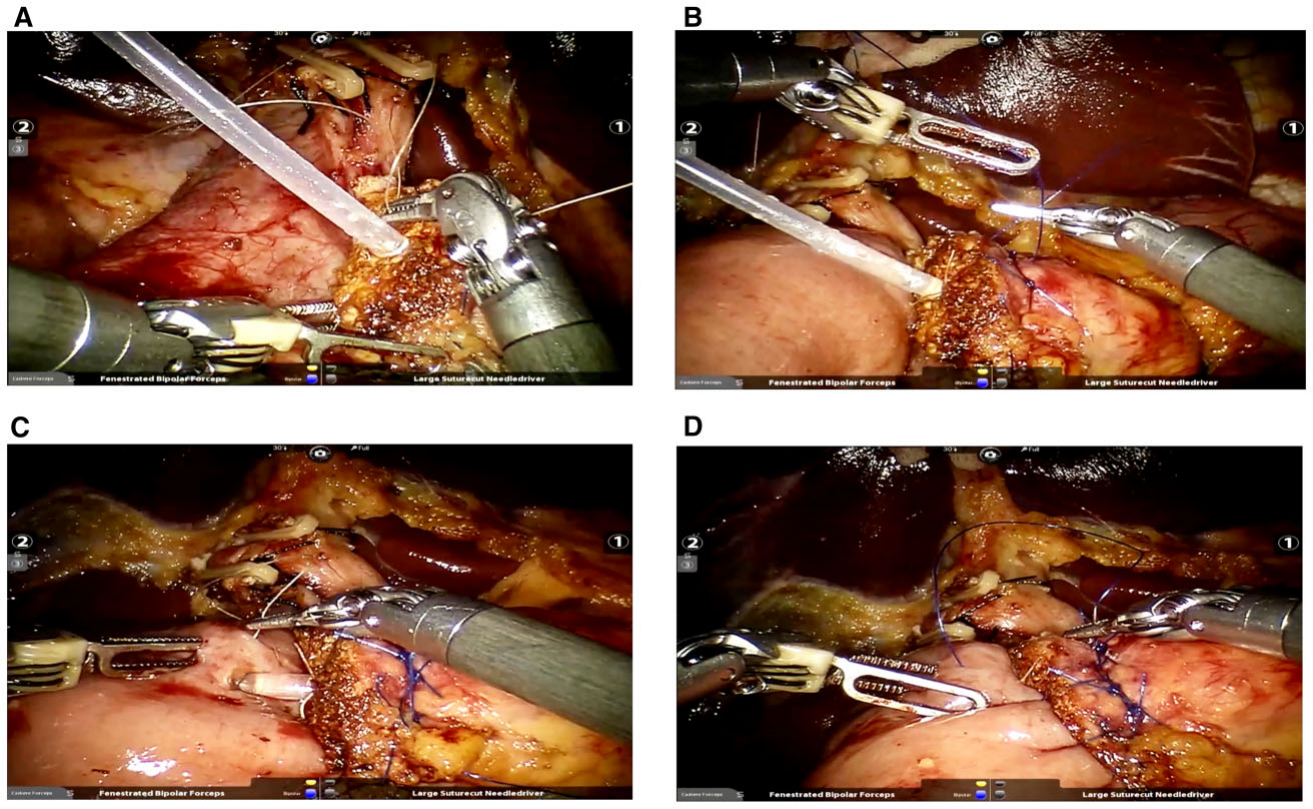
The procedure of “stent-bridged” pancreatojejunostomy. (A) The stent is inserted into the pancreatic duct and fixed with figure-of-8 suturing. (B) The posterior external wall is anastomosed by three overlapping transpancreatic U-sutures. (C) The stent is inserted into the downstream jejunum via the enterotomy and a purse-string suture of the jejunal seromuscular layer around the stent was made. (D) Anterior external wall is anastomosed by continuous suture.

- Kocher maneuver: After dividing the gastrocolic ligament, the second and third parts of the duodenum and the pancreatic head were isolated until exposure of the inferior vena cava, abdominal aorta, left renal vein, and root of the superior mesenteric artery (SMA).
- Dissection of SMV: SMV was dissected along the inferior and posterior to the pancreatic neck. The right gastroepiploic vessels and Henle’s trunk were ligated and divided.
- Transection of the distal stomach and lymphadenectomy: The stomach was transected at the intersection between the gastric antrum and the body by using a stapler. Regional lymphadenectomy was performed to expose the common hepatic artery, proper hepatic artery, right gastric artery (RGA), and gastroduodenal artery (GDA). The RGA and GDA were then ligated and/or clipped and divided. The PV was dissected at the superior to the pancreatic neck.
- Transection of the pancreatic neck: The pancreatic neck was transected anterior to the SMV and PV by using electrocauterization.
- Division of the proximal jejunum, uncinate process, and common bile duct, and resection of the gallbladder: After mobilizing the Treitz ligament, the proximal jejunum was pulled to the right side through the root of the mesocolon and then was transected at ∼15 cm from the Treitz ligament by using a stapler. The uncinate process was divided upwardly by using the harmonic scalpel. The vessels entering the pancreas from the right side of the SMV and SMA were clipped and divided. The common bile duct was transected and the gallbladder was removed.
- Reconstruction of the pancreatojejunostomy (PJ): The following two types of the PJ were used: (a) end-to-side mucosa-to-mucosa PJ (Figure 3); and (b) the “stent-bridged” PJ (Figure 4).
- Reconstruction of the cholangiojejunostomy (CJ): The CJ was performed by using continuous suture for the dilated bile duct or interrupted suture for the non-dilated bile duct.
- Reconstruction of the gastrojejunostomy (GJ): The GJ was completed by using totally robotic-assisted double-layered suturing.
- Drainage placement and closure of the wound: The drainage tubes were routinely placed close to the PJ and CJ, respectively.

Steps (i)–(v) were defined as the resection procedure and Steps (vi)–(viii) were defined as the reconstruction procedure. Notably, the order of resection (Steps (i)–(v)) could be adjusted individually by using the “easy-first” strategy according to the size, location, and vascular relationship of the tumor and the presence of chronic pancreatitis. On the other hand, the order of reconstruction (Steps (vi)–(viii)) was basically fixed.

### Data collection and definition

The resection procedure time was defined as the interval from the division of the gastrocolic ligament to the removal of the pancreatoduodenal specimen. The reconstruction procedure time was defined as the interval from the beginning of the PJ to the end of the GJ.

The serial number for each patient was given in the order of the surgery date. Post-operative complications were evaluated according to the Clavien–Dindo (CD) classification [14] and complications with a severity of ≥Grade III were defined as major complications. POPF was determined according to the 2016 International Study Group of Pancreatic Surgery (ISGPS) definition and grading of the POPF [15]. The CR-POPF includes Grade B and Grade C POPF.

### Statistical analysis

Statistical analyses were performed using SPSS version 24.0 software (IBM, Inc., Armonk, NY, USA) and R version 4.0.0 (http://www.Rproject.org). The categorical variables are presented as frequencies with percentages and the continuous variables are presented as medians with interquartile range (IQR). Differences between categorical variables were analysed by using the chi-square test or Fisher’s exact test. Differences between continuous variables were analysed by using the Kruskal–Wallis test. The learning curve was described using a cumulative sum (CUSUM) curve[10]. Two-tailed *P *<* *0.05 was considered statistically significant.

## Results

### Clinicopathological features and outcomes of patients undergoing RPD

A total of 160 consecutive patients undergoing RPD were enrolled and divided into eight groups of 20 cases in the order of the surgery date. The clinicopathological features of patients were summarized and compared (Table 1). Eighty-six patients (53.1%) were male and the median age was 60 (IQR, 49–68) years. The median body mass index (BMI) was 22.5 (IQR, 20.2–24.6) kg/m^2^. Among 160 patients, 23 (14.4%) had a history of previous abdominal surgery; 31 (19.4%) were ampullary carcinomas, 30 (18.8%) pancreatic ductal adenocarcinomas, 27 (16.9%) pancreatic cystic neoplasms, 26 (16.3%) neuroendocrine tumors, 23 (14.4%) duodenal or duodenal papillary carcinomas, 9 (5.6%) chronic pancreatitis, 6 (3.8%) distal cholangiocarcinomas, and 8 (5.0%) other pathologies. There were no significant differences between the eight groups, except for the sex, American Society of Anesthesiologists (ASA) classification, and tumor size (Table 1).

**Table 1.** Comparison of baseline clinicopathological characteristics of patients undergoing RPD

Feature	Total	Group (Cases)								
		1 (1–20)	2 (21–40)	3 (41–60)	4 (61–80)	5 (81–100)	6 (101–120)	7 (121–140)	8 (141–160)	***P*-value** ^a^
Age, years, median (IQR)	60 (49–68)	56 (45–68)	58 (50–67)	54 (47–70)	63 (46–69)	61 (41–67)	59 (50–67)	64 (60–75)	54 (45–63)	0.227^b^
Sex, male, *n* (%)	86 (53.8%)	10 (50.0%)	5 (25.0%)	9 (45.0%)	14 (70.0%)	9 (45.0%)	15 (75.0%)	10 (50.0%)	14 (70.0%)	0.025^c^
BMI, kg/m^2^, median (IQR)	22.5 (20.2–24.6)	24.4 (20.6–26.1)	22.8 (20.8–23.7)	21.6 (19.1–23.8)	22.4 (20.8–24.0)	22.4 (20.1–24.7)	23.4 (19.4–26.0)	22.4 (19.9–24.3)	22.3 (19.1–24.2)	0.649^b^
ASA classification ≥III, *n* (%)	58 (36.3%)	4 (20.0%)	2 (10.0%)	3 (15.0%)	2 (10.0%)	10 (50.0%)	9 (45.0%)	15 (75.0%)	13 (65.0%)	<0.001
Abdominal surgery history, *n* (%)	23 (14.4%)	0 (0%)	2 (10.0%)	2 (10.0%)	2 (10.0%)	2 (10.0%)	4 (20.0%)	7 (35.0%)	4 (20.0%)	0.084
Diabetes, *n* (%)	23 (14.4%)	5 (25.0%)	3 (15.0%)	3 (15.0%)	3 (15.0%)	3 (15.0%)	1 (5.0%)	3 (15.0%)	2 (10.0%)	0.852
Preoperative biliary drainage, *n* (%)	56 (35.0%)	8 (40.0%)	8 (40.0%)	7 (35.0%)	7 (35.0%)	9 (45.0%)	6 (30.0%)	6 (30.0%)	5 (25.0%)	0.935
Hemoglobin level, g/L, median (IQR)	122 (109–135)	131 (113–137)	121 (117–135)	113 (104–128)	116 (108–130)	128 (109–137)	129 (115–140)	120 (105–131)	119 (108–142)	0.182^b^
Albumin level, median (IQR)	38.5 (36.0–40.9)	39.1 (36.0–45.3)	38.5 (36.5–40.8)	39.8 (35.7–43.9)	37.8 (34.5–39.8)	38.2 (35.6–41.7)	37.5 (36.1–39.1)	38.8 (37.7–40.3)	38.8 (37.6–40.9)	0.431^b^
Tumor size, cm, median (IQR)	2.0 (1.0–3.0)	1.5 (0.5–3.5)	1.6 (0.6–2.9)	1.6 (0.9–2.5)	2.1 (1.5–3.2)	1.7 (1.0–2.5)	2.5 (1.1–3.5)	2.0 (1.5–3.2)	3.0 (2.0–4.4)	0.040^b^
Pancreatic adenocarcinoma, *n* (%)	30 (18.8%)	4 (20.0%)	3 (15.0%)	3 (15.0%)	3 (15.0%)	2 (10.0%)	4 (20.0%)	6 (30.0%)	5 (25.0%)	0.857
Pancreatic duct ≥3 mm, *n* (%)	110 (68.8%)	12 (60.0%)	12 (60.0%)	15 (75.0%)	17 (85.0%)	12 (60.0%)	14 (70.0%)	15 (75.0%)	13 (65.0%)	0.594
Firm pancreatic texture, *n* (%)	63 (39.4%)	6 (30.0%)	8 (40.0%)	6 (30.0%)	8 (40.0%)	9 (45.0%)	10 (50.0%)	6 (30.0%)	10 (50.0%)	0.736^c^

a Fisher’s exact test.

b Kruskal–Wallis test.

c Chi-square test.

RPD, robotic-assisted pancreatoduodenectomy; IQR, interquartile range; BMI, body mass index; ASA, American Society of Anesthesiologists.

### Surgical details and post-operative outcomes of patients undergoing RPD

Surgical details and post-operative outcomes of patients undergoing RPD are summarized in Table 2. The median times of the resection and reconstruction procedures was 206 (IQR, 170–270) min and 125 (IQR, 110–144) min, respectively. The median estimated intraoperative blood loss (IBL) was 50 (IQR, 50–100) mL. Two cases (1.3%) were converted into open surgery because of close abutment between the tumor and the vessels. R0 resection was achieved in 159 (99.4%) patients with a median total number of lymph nodes examined (TNLE) of 10 (IQR, 6–13).

**Table 2.** Comparison of perioperative outcomes of patients undergoing RPD

Feature	Total	Group (Cases)								
		1 (1–20)	2 (21–40)	3 (41–60)	4 (61–80)	5 (81–100)	6 (101–120)	7 (121–140)	8 (141–160)	***P*-value** ^a^
Conversion, *n* (%)	2 (1.3%)	0 (0%)	0 (0%)	1 (5.0%)	0 (0%)	0 (0%)	1 (5.0%)	0 (0%)	0 (0%)	1.000
R0 resection, *n* (%)	159 (99.4%)	20 (100.0%)	20 (100.0%)	19 (95.0%)	20 (100.0%)	20 (100.0%)	20 (100.0%)	20 (100.0%)	20 (100.0%)	1.000
Resection time, min, median (IQR)	206 (170–270)	309 (248–362)	188 (176–226)	189 (154–209)	199 (164–265)	199 (165–218)	224 (164–264)	184 (163–281)	201 (155–271)	0.001^b^
Reconstruction time, min, median (IQR)	125 (110–144)	163 (139–205)	132 (122–154)	123 (110–138)	112 (97–120)	123 (106–134)	132 (113–158)	117 (104–139)	116 (107–125)	<0.001^b^
IBL, mL, median (IQR)	50 (50–100)	100 (63–100)	50 (50–100)	50 (50–50)	50 (50–100)	50 (50–50)	50 (50–88)	50 (50–50)	55 (50–80)	0.002^b^
Blood transfusion, *n* (%)	30 (18.7%)	2 (10.0%)	7 (35.0%)	3 (15.0%)	3 (15.0%)	5 (25.0%)	2 (10.0%)	4 (20.0%)	4 (20.0%)	0.562
TNLE, median (IQR)	10 (6–13)	8 (5–11)	9 (6–14)	10 (9–16)	11 (6–14)	12 (8–13)	7 (4–12)	9 (5–19)	9 (6–11)	0.169^b^
Major complications^d^, *n* (%)	62 (38.8%)	5 (25.0%)	6 (30.0%)	9 (45.0%)	8 (40.0%)	13 (65.0%)	7 (35.0%)	7 (35.0%)	7 (35.0%)	0.272^c^
Reoperation, *n* (%)	5 (3.1%)	1 (5.0%)	1 (5.0%)	1 (5.0%)	2 (10.0%)	0 (0%)	0 (0%)	0 (0%)	0 (0%)	0.782
CR-POPF, *n* (%)	40 (25.0%)	5 (25.0%)	4 (20.0%)	5 (25.0%)	5 (25.0%)	12 (60.0%)	4 (20.0%)	3 (15.0%)	2 (10.0%)	0.033
Grade B	40 (25.0%)	5 (25.0%)	4 (20.0%)	5 (25.0%)	5 (25.0%)	12 (60.0%)	4 (20.0%)	3 (15.0%)	2 (10.0%)	
Grade C	0 (0%)	0 (0%)	0 (0%)	0 (0%)	0 (0%)	0 (0%)	0 (0%)	0 (0%)	0 (0%)	
30-day mortality, *n* (%)	1 (0.6%)	0 (0%)	0 (0%)	0 (0%)	1 (5.0%)	0 (0%)	0 (0%)	0 (0%)	0 (0%)	1.000
Post-operative LOS, days, median (IQR)	16 (11–24)	12 (10–18)	16 (12–23)	15 (12–25)	14 (9–23)	21 (11–39)	19 (12–24)	18 (11–33)	15 (10–20)	0.173^b^
30-day readmission, *n* (%)	17 (10.6%)	1 (5.0%)	3 (15.0%)	2 (10.0%)	3 (15.0%)	1 (5.0%)	3 (15.0%)	1 (5.0%)	3 (15.0%)	0.822

a Fisher’s exact test.

b Kruskal–Wallis test.

c Chi-square test.

d Severity ≥Clavien–Dindo classification Grade III.

RPD, robotic-assisted pancreatoduodenectomy; IQR, interquartile range; IBL, intraoperative blood loss; TNLE, total number of lymph nodes examined; CR-POPF, clinically relevant post-operative pancreatic fistula; LOS, length-of-stay; NA, not available.

Forty patients (25.0%) developed CR-POPF; all of these patients had Grade B CR-POPF. Sixty-two patients (38.8%) developed post-operative major complications (CD classification Grade III–V). The median post-operative length-of-stay (LOS) was 16 (IQR, 11–24) days.

Reoperation was performed in five cases (3.1%), including three cases of post-operative hemorrhage and two cases of intestinal obstruction. One patient died within 30 days post-operatively (30-day mortality rate, 0.6%); this patient suffered from uncontrollable intra-abdominal infections leading to respiratory failure after reoperation.

### Learning curves of resection and reconstruction procedures in RPD

**Figure 5.**
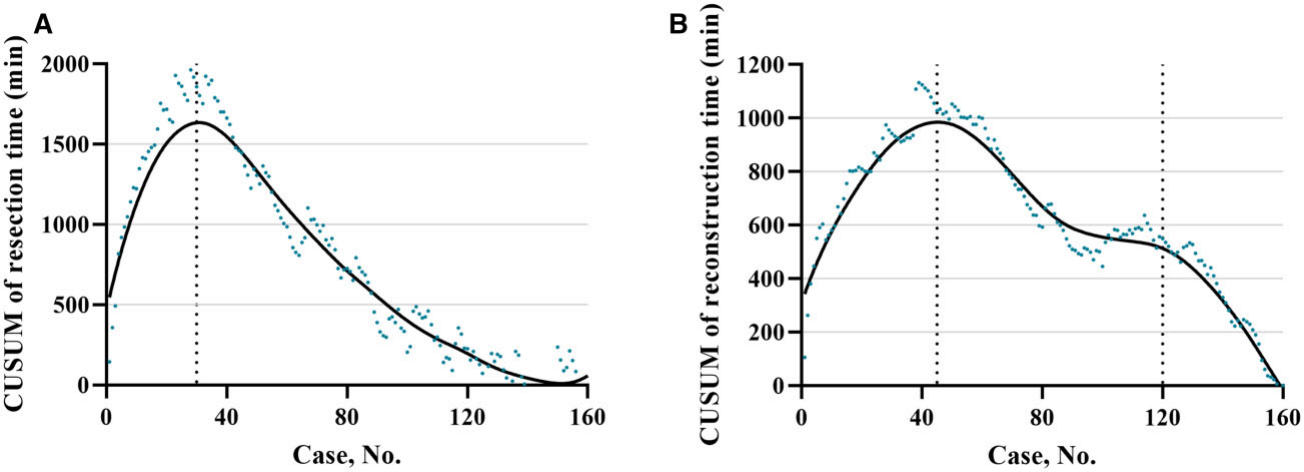
Learning curve of robotic-assisted pancreatoduodenectomy. (A) The inflection point of the resection procedure time is around Case 30. (B) The inflection point of the reconstruction procedure time is around Case 45.

The learning curves of the resection procedure (Figure 5A) and the reconstruction procedure (Figure 5B) were analysed. The results showed that the resection time decreased significantly after 30 cases (median, 284 vs 195 min; *P *<* *0.001) and the reconstruction time deseased significantly after 45 cases (median, 138 vs 120 min; *P *<* *0.001).

**Figure 6.**
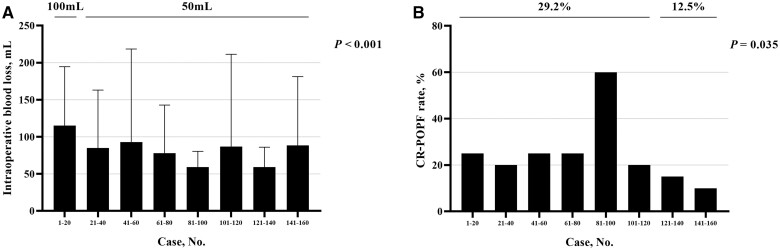
Improvement of perioperative outcomes in patients undergoing robotic-assisted pancreatoduodenectomy. (A) The intraoperative estimated blood loss deseased after 20 cases. (B) The incidence of CR-POPF declined after 120 cases. CR-POPF, clinically relevant post-operative pancreatic fistula.

**Figure 7.**
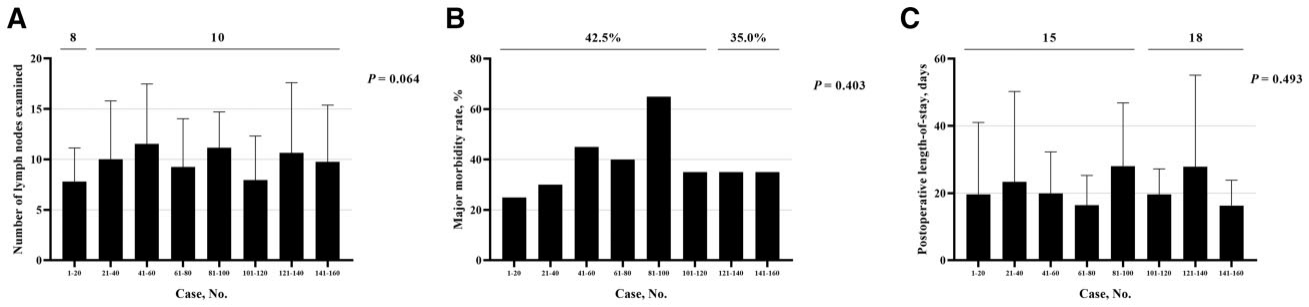
Comparison of (A) total number of lymph nodes examined, (B) post-operative major complication rate, and (C) post-operative length-of-stay between different groups.

The estimated IBL decreased from 100 (IQR, 63–100) mL (Cases 1–20) to 50 (IQR, 50–78) mL (Cases 21–160) (*P *<* *0.001; Figure 6A). The incidence of CR-POPF in the last 40 cases was significantly declined compared with that in the first 120 cases (29.2% vs 12.5%, *P *=* *0.035; Figure 6B), while there were no significant differences in the size of the pancreatic duct (≥3 mm, 68.3% vs 70.0%, *P *=* *0.844) or the pancreatic texture (firm, 39.2% vs 40.0%, *P *=* *0.926) between the two groups. However, there was no statistical difference in the TNLE (median, 8 vs 10; *P *=* *0.064; Figure 7A), post-operative major morbidity rate (42.5% vs 35.0%, *P *=* *0.403; Figure 7B), or post-operative LOS (median, 15 vs 18 days; *P *=* *0.493; Figure 7C) between the groups.

Because the CR-POPF rate decreased significantly after 120 RPD cases, the operation time before and after 120 RPD cases was further evaluated. The results showed that the reconstruction time (median, 128 vs 116 min; *P *=* *0.004) decreased significantly after 120 RPD cases, but there was no significant reduction in the resection time (median, 208 vs 185 min; *P *=* *0.291) after 120 RPD cases.

## Discussion

The robotic-assisted surgical system has been gaining popularity worldwide since it was applied in pancreatic surgery in 2002 [16]. Because the robotic-assisted surgical system has the advantages of 3D vision and a stable manipulator, the learning curve of RPD was theoretically shorter than that of LPD [17]. However, due to the lack of force feedback, the surgeon needs to overcome this shortcoming by learning “visual feedback” after a number of cases [10]. So far, four studies have reported the learning curve of RPD with more than 150 cases in a single center [9–12]. A retrospective analysis of 450 RPD cases showed that there were three phases and two important inflexion points in the RPD learning curve (100 cases and 250 cases) [10]. Another analysis of 200 RPD cases reported that the inflection points were ∼80 cases and ∼140 cases [11]. Another report of 100 RPD cases showed that the learning curve of RPD was completed after 40 cases [18]. In these studies, RPD was completed by more than one surgeon. The variations among operators would sacrifice the accuracy of its learning curve.

In this study, we analysed the learning curves of the two major procedures of RPD, i.e. the resection procedure and the reconstruction procedure, by a single surgeon who had the experience of finishing more than 100 cases of open PD but no experience of laparoscopic PD before embarking on RPD. It was found that the inflection points in the resection and reconstruction procedures were ∼30 cases and ∼45 cases, respectively. In contrast, the CR-POPF rate did not decrease significantly until 120 RPD cases. It indicated that 120 cases were necessary for the technical maturation of RPD in an individual surgeon.

The operation time reflects the fluency of the surgery. In order to shorten the operation time, the “nine-step” procedure and “easy-first” strategy of RPD were proposed after a number of cases were handled. Frequent interchanges of instruments are an adverse factor in prolonging the operation time of RPD. The previous literature reported that all blood vessels were sutured after excision of the specimen for the purpose of shortening the operation time [10]. However, this maneuver is potentially risky and difficult to replicate because negligence in suturing some blood vessels may increase the risk of massive intraoperative or post-operative bleeding [10]. We found a significant reduction in the resection time after 30 RPD cases, which was mainly due to the optimization of the resection process (“nine-step” procedure and “easy-first” strategy) and the reduced frequency of instruments interchanges.

The risk of post-operative morbidities was determined by the quality of the reconstruction, especially the quality of the PJ. We found that the reconstruction time was mainly affected by the PJ time. After 45 cases of RPD, the reconstruction procedure time was significantly shortened. The method of PJ in this study included mucosa-to-mucosa PJ and “stent-bridged” PJ. “Stent-bridged” PJ was frequently used in the RPD cases with small pancreatic ducts and more mucosa-to-mucosa PJ were adopted in the RPD cases with normal or dilated pancreatic ducts. The method of CJ depended on the diameter of the bile duct. Gastrojejunostomy was generally completed by totally robotic-assisted double-layered suturing, which helped to reduce the risk of post-operative anastomotic bleeding.

The CR-POPF rate reported in this study was relatively higher than those reported by other studies of RPD [10, 11]. The reason might be the relatively higher proportion of pathologies in this study, including ampullary carcinoma, duodenum carcinoma, pancreatic cystic neoplasms, and neuroendocrine tumors. These pathologies might increase the risk of CR-POPF because of the soft pancreatic parenchyma and the small size of the main pancreatic duct [19]. Besides, the lack of force feedback during suturing might have reduced the quality of the PJ in the early RPD cases. However, the lack of force feedback could be overcome by learning “visual feedback,” which was helpful in decreasing the risk of CR-POPF significantly.

The current study had several limitations. First, it was a retrospective study and had inevitable biases. Second, all RPDs in this study were performed by using the da Vinci Si Surgical System, which may lead to drawbacks such as a longer operation time compared with the latest Xi surgical system [20]. Third, the study was conducted by a single surgeon at a specific hospital, which may limit the generalizability of the findings to other centers or surgeons. Further research involving multiple centers and surgeons would be beneficial to validate these results. Besides, this study excluded RPDs with vascular resection or additional organ resection. The efficacy of RPD combined with vascular resection and the long-term outcome in malignant diseases need to be explored by using larger multi-institutional prospective trials.

## Conclusions

This study revealed that RPD was a safe and feasible approach for the selected patients with acceptable post-operative morbidity and mortality. The resection time, reconstruction time, and CR-POPF rate decreased after 30, 45, and 120 cases, respectively.
